# Nanovoid formation mechanism in nanotwinned Cu

**DOI:** 10.1186/s11671-024-03984-z

**Published:** 2024-03-12

**Authors:** Cuncai Fan, Haiyan Wang, Xinghang Zhang

**Affiliations:** 1 Department of Mechanical Engineering, City University of Hong Kong, Kowloon, Hong Kong China; 2 School of Materials Engineering, Purdue University, West Lafayette, IN 47907 USA; 3 School of Electrical and Computer Engineering, Purdue University, West Lafayette, IN 47907 USA

**Keywords:** Magnetron sputtering, Nanotwinned metals, Nanovoids, Epitaxial film

## Abstract

Nanotwinned metals have been intensely investigated due to their unique microstructures and superior properties. This work aims to investigate the nanovoid formation mechanism in sputter-deposited nanotwinned Cu. Three different types of epitaxial or polycrystalline Cu films are fabricated by magnetron sputtering deposition technique. In the epitaxial Cu (111) films deposited on Si (110) substrates, high fractions of nanovoids and nanotwins are formed. The void size and density can be tailored by varying deposition parameters, including argon pressure, deposition rate, and film thickness. Interestingly, nanovoids become absent in the polycrystalline Cu film deposited on Si (111) substrate, but they can be regained in the epitaxial nanotwinned Cu (111) when deposited on Si (111) substrate with an Ag seed layer. The nanovoid formation seems to be closely associated with twin nucleation and film texture. Based on the comparative studies between void-free polycrystalline Cu films and epitaxial nanotwinned Cu films with nanovoids, the underlying mechanisms for the formation of nanovoids are discussed within the framework of island coalescence model.

**Graphical abstract:**

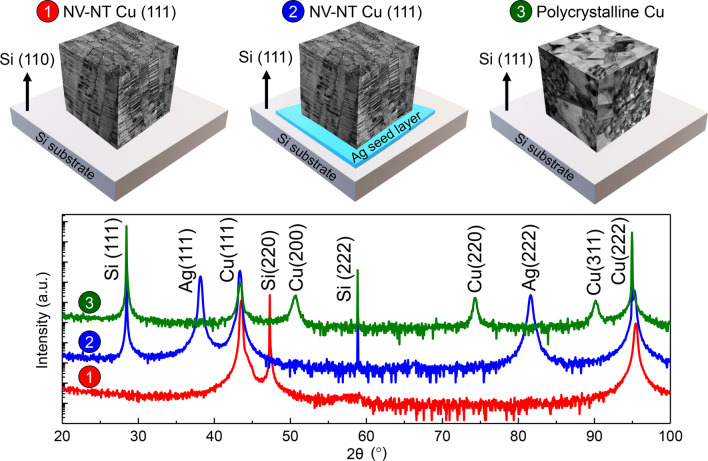

## Introduction

As a typical method of physical vapor deposition (PVD), magnetron sputtering technique is attractive to industry for fabricating various metallic films and coatings with unique microstructures and properties [1]. Sputtering deposition normally takes place in a vacuum chamber where the atomic flux of source material is transferred from the target (cathode) to the substrate (anode) [2]. Since this process is largely nonequilibrium, involving the sputtering of a target by energetic ions (e.g., Ar ions) and the condensation of a vapor into a solid, as-deposited films can be far away from their energetic minimums [3]. As a result, the microstructure of sputter-deposited coatings is characterized by a large number of defects, such as grain boundaries and voids [4]. For instance, previous studies have revealed that the polycrystalline metallic films synthesized by sputtering at low temperatures consist of fine grains ranging from several to tens of nanometers in size [5]. Especially, in sputter-deposited films of face-centered-cubic (FCC) metals with a low stacking fault energy, such as copper (Cu), silver (Ag), and 330 steel [6–8], a considerable fraction of nanoscale growth twins can be formed. The twin structures are bounded by a special type of high-angle grain boundary, the coherent twin boundary (CTB) that stores a minimal boundary energy and renders nanotwinned (NT) metals superior physical and mechanical properties [9, 10]. Compared with nanograined and coarse-grained counterparts, the NT metals exhibit combinations of high electrical conductivity [11, 12], good thermal stability [13–15], outstanding radiation tolerance [16–19], as well as high strength and ductility [20, 21]. Recently, it has been found that the mechanical properties of NT metal films can be enhanced further by introducing nanovoids [22, 23]. Our previous studies also found that preexisting nanovoids can improve the radiation tolerance of NT metals [24–26], as they can act as the effective sinks for radiation-induced defects [27, 28]. Although the effects of nanovoids on mechanical and radiation properties have been systematically investigated, the underlying mechanism of nanovoid formation in NT films is still unclear. To advance the applications of NT metals in surface and coatings technology and to extend our understandings on the sputtered nanostructured materials, it is warranted to investigate how nanovoids form and evolve during film growth.

This work focuses on the nanovoid evolution in sputter-deposited NT and polycrystalline Cu films. The variations of nanovoid size and density with increasing Ar working pressure, deposition rate, and film thickness were systematically investigated. Experimental results revealed that the nanovoid formation is closely associated with twin nucleation and film texture, hence this study provides new insights into design and fabrication of NT metallic films with nanovoids.

## Experimental

High purity (99.995%) Cu thin films were deposited onto the HF-etched silicon (Si) wafers at room temperature using a custom-designed direct current magnetron sputtering system. Prior to depositions, the main chamber was pumped to a typical base pressure < 8 × 10^−8^ torr. Sputtered films can be divided into three types according to the variations of deposition condition, orientation of Si substrate, and the seed layer on Si substrates. Type 1 films were directly deposited on Si (110) substrates. They include three subgroups with different Ar working pressures *P*_*Ar*_, deposition rates *R*_*Dep*._, and film thicknesses *T*_*Flim*_, as summarized in Table 1. In contrast, Types 2 and 3 refer to the Cu films deposited on Si (111) substrates, respectively, without and with an Ag seed layer (~ 200 nm thick); they have the same Ar working pressure (~ 2.6 mtorr), deposition rate (~ 1 nm/s), and total film thickness (~ 2 μm). The deposition rate in each sample was controlled by changing Ar working pressure and sputtering power.

**Table 1:** Sputtering conditions, microstructures, and residual stress of the Type 1 NT–NV Cu (111) films grown on Si (110) substrates

Sample label	Deposition conditions			Microstructures				Film residual stress *σ* (MPa)
	P_Ar_ (mtorr)	R_Dep._ (nm/s)	T_Flim_ (μm)	D (nm)	t (nm)	V (nm)	ρ_V_ (10^–4^ nm^2^)	
a1_1.7 mtorr	1.7	0.6	2.2	120 ± 27	7 ± 4	6 ± 2	3.4 ± 0.5	581 ± 1
a2_3.6 mtorr	3.6	0.6	2.2	94 ± 21	6 ± 2	5 ± 2	6.6 ± 0.4	604 ± 5
a3_5.5 mtorr	5.5	0.6	2.1	94 ± 22	8 ± 4	5 ± 1	12.9 ± 1.0	528 ± 4
b1_0.2 nm/s	2.6	0.2	2.0	123 ± 16	7 ± 6	7 ± 3	2.1 ± 0.2	836 ± 40
b2_1.3 nm/s	2.6	1.3	2.3	120 ± 19	8 ± 5	5 ± 1	5.5 ± 0.4	654 ± 30
b3_2.9 nm/s	2.6	2.9	2.1	115 ± 20	5 ± 3	6 ± 2	8.5 ± 0.7	822 ± 63
c1_1.2 μm	2.6	0.6	1.2	91 ± 14	5 ± 3	5 ± 3	8.8 ± 0.5	690 ± 60
c2_2.5 μm	2.6	0.6	2.4	115 ± 19	6 ± 2	6 ± 2	5.0 ± 0.4	561 ± 2
c3_6.5 μm	2.6	0.6	6.5	180 ± 24	26 ± 22	14 ± 5	0.9 ± 0.1	424 ± 6

*P*_*Ar*_ Ar pressure, *R*_*Dep.*_ deposition rate, *T*_*Flim*_ film thickness, *D* domain size, *t* twin spacing, *V* void size, *ρ*_*V*_ void density, *σ* film residual stress

The X-ray diffraction (XRD) analyses of as-deposited films were performed by a Panalytical Empyrean X'pert PRO MRD X-ray diffractometer with a Cu Kα1 source. The film surface was characterized by an FEI Nova NanoSem 450 scanning electron microscope (SEM) operated at 20 kV. Plan-view and cross-section transmission electron microscope (TEM) specimens were prepared by polishing, dimpling, and low energy Ar ion milling. All the TEM specimens were subsequently examined by an FEI Talos 200X TEM operated at 200 kV. Besides, the Si substrate radii, $R_{0}$ and $R_{1}$ before and after film deposition, were measured using a profilometer, and film residual stress *σ* was calculated based on the Stoney formula [29]

1 $$\sigma = \frac{{M_{s} T_{Si}^{2} }}{{6T_{Film} }}\left( {\frac{1}{{R_{1} }} - \frac{1}{{R_{0} }}} \right)$$

where $T_{Si}$ is the Si substate thickness (~ 500 μm), and $M_{s}$ is the biaxial modulus of the substrate, ~ 217 GPa for Si (110) [30].

## Results

### Type 1: nanovoid–nanotwinned Cu (111) directly deposited on Si (110) in different deposition conditions

**Fig. 1:**
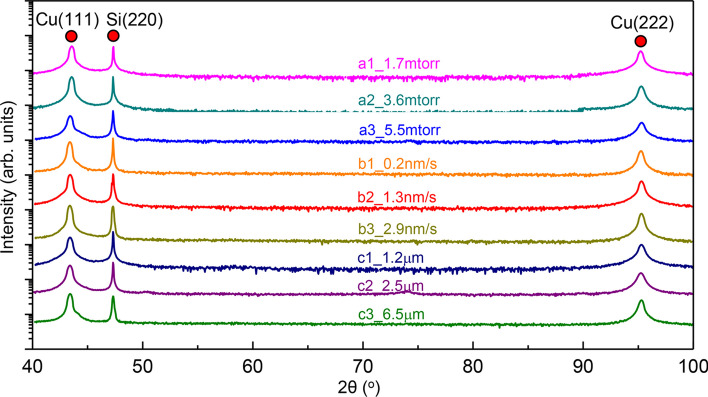
(Color online) The XRD 2*θ*-scan profiles of Cu films directly deposited on Si (110) substrates. The existence of only Cu (111) and (222) suggests the formation of epitaxial films along growth direction. The spectra correspond to the samples synthesized with increasing argon working pressure from 1.7 to 5.5 mtorr (a1)–(a3), increasing deposition rate from 0.2 to 2.9 nm/s (b1)–(b3), and increasing film thickness from 1.2 to 6.5 μm (c1)–(c3). The samples are summarized in Table 1

**Fig. 2:**
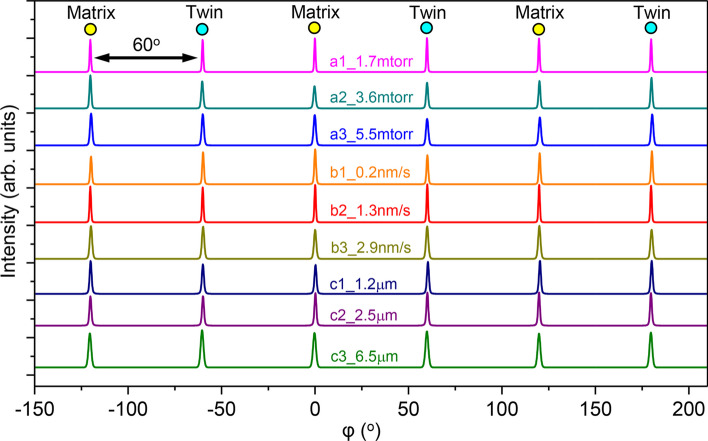
(Color online) The XRD *φ*-scan profiles of Cu {111} with a six-fold symmetry indicating the formation of high-density growth twins in as-deposited films

Following our previous studies [24, 25], we first investigated the effects of sputtering condition parameters on the evolutions of texture of Cu films directly deposited on Si (110) substrates. Figure 1 compiles the normal XRD 2*θ*-scan profiles of as-deposited films. Apart from Si (220), the X-ray spectra only show two strong peaks, namely Cu (111) and Cu (222), indicating the formation of epitaxial Cu (111) films in all cases, regardless of the variation of Ar pressure from 1.7 to 5.5 mtorr, the increasing deposition rate from 0.2 to 2.9 nm/s, and the increasing film thickness from 1.2 to 6.5 μm. The corresponding XRD *φ*-scan profiles of Cu {111} in Fig. 2 show strong peaks with a six-fold symmetry. These peaks must arise from two sets of variants with a 60° rotation angle along the out-of-plane direction, that is the Cu <111> crystallographic direction, indicating the formation of a significant fraction of growth twins in sputtered Cu films.

**Fig. 3:**
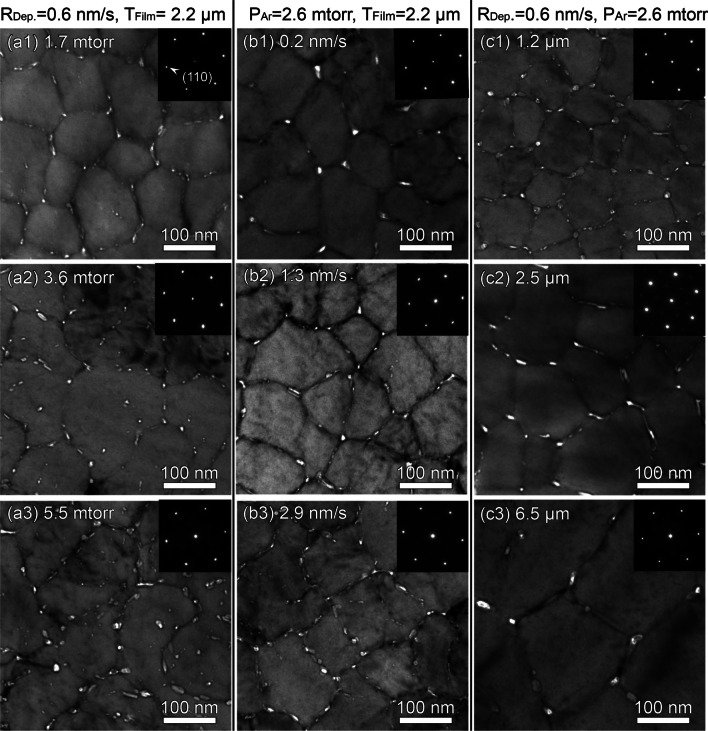
Plan-view TEM micrographs displaying abundant nanovoids that are mostly distributed along domain boundaries. The inset SAD patterns clearly show single crystal-like diffraction along the Cu <111> zone axis

**Fig. 4:**
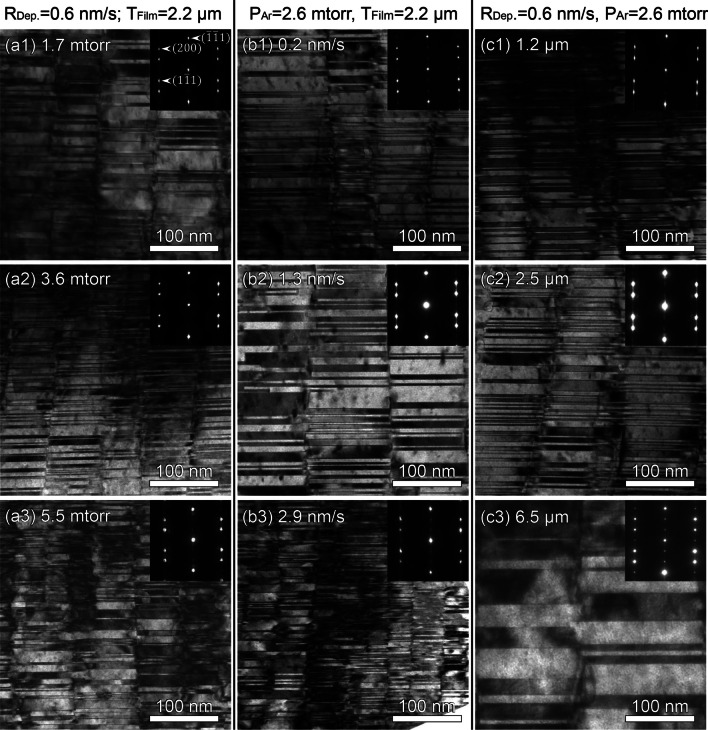
Cross-section TEM micrographs captured from Cu <110> zone axis, revealing columnar domains and high-density growth twins in epitaxial Cu (111) films. The inset SAD patterns confirm the formation of twin structures

The plan-view TEM micrographs of as-deposited Cu films in Fig. 3 show that the films contain polygonal domains, and there are abundant nanovoids randomly distributed along domain boundaries. Moreover, the inset selected area diffraction (SAD) patterns demonstrate the formation of single crystal-like grains oriented along <111> direction, consistent with the XRD profiles presented in Fig. 1. The cross-section TEM micrographs in Fig. 4 show columnar domains consisting of high-density CTBs. These boundaries appear every few nanometers and are normal to the film growth direction. The inset SAD patterns confirm the formation of growth twins, also in good agreement with the *φ*-scan profiles in Fig. 2. Since the NT samples have abundant nanovoids, hereafter we will refer to them as nanovoid–nanotwinned (NV–NT) Cu.

**Fig. 5:**
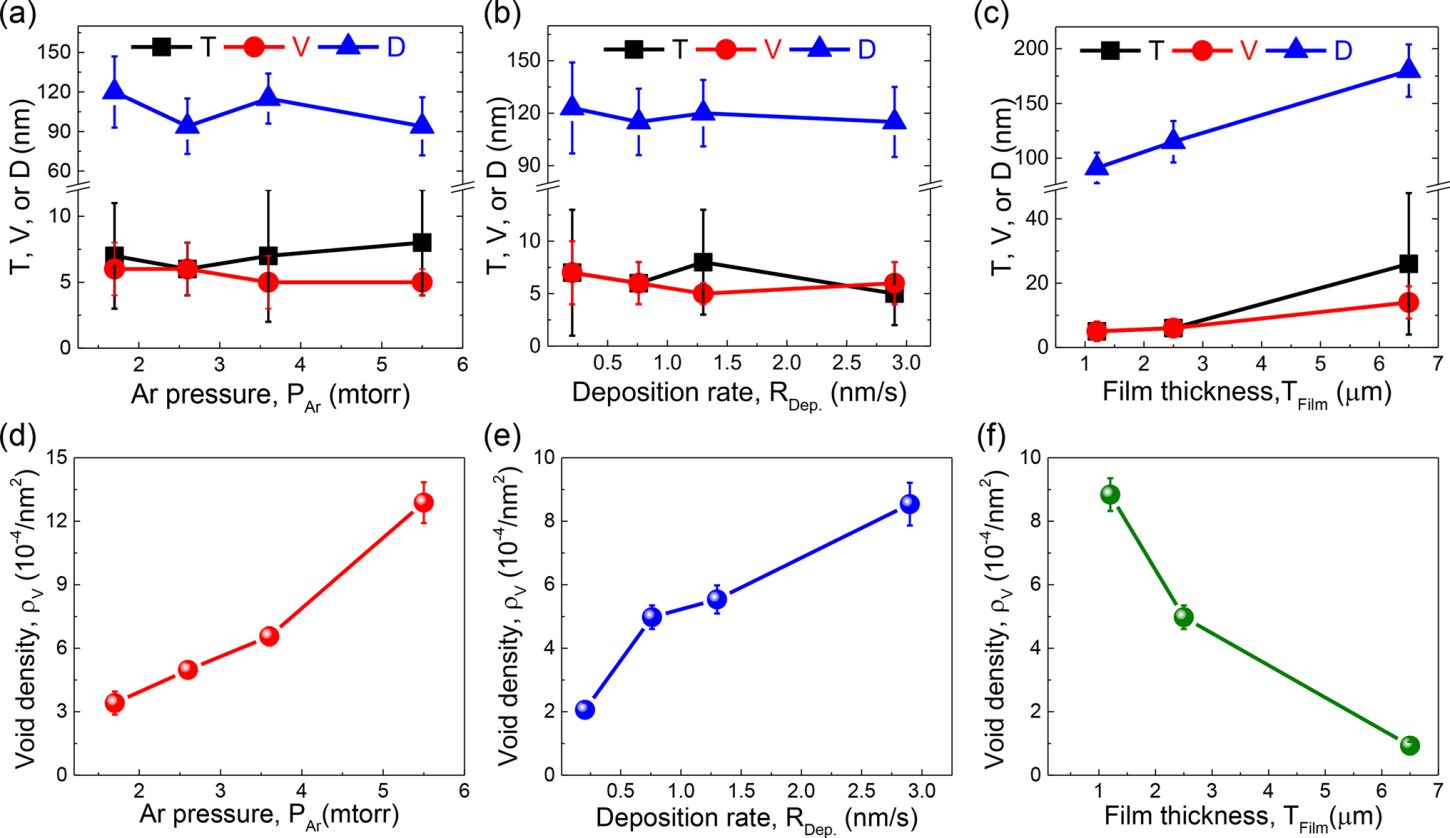
**a**–**c** Variations of twin spacing T, void size V, and domain size D with increasing Ar pressure P_Ar_, deposition rate R_Dep._, and film thickness T_Flim_. **d**–**f** Void density ρ_V_ plotted as a function of deposition parameters

It seems that varying deposition parameters can hardly change the texture of NV–NT Cu film. Other microstructure features indeed vary, including twin spacing, void size and density, as well as domain size. As shown in Fig. 5a–c, the twin spacing t, void size V, and domain size D fluctuates slightly with varying Ar pressure P_Ar_ and deposition rate R_Dep._. However, t, V and D all enlarge with increasing film thickness T_Flim_. This variation could be caused by annealing, as it takes a longer time to deposit a thicker film when heat cannot be transferred instantly from Si wafer. It is worth noting that the void size V is comparable to the twin spacing t. This aspect will be discussed later in more detail. It is also noted that the void density, ρ_V_ changes prominently with deposition conditions. As shown in Fig. 5d–f, ρ_V_ increases with increasing P_Ar_ and R_Dep._, but it decreases with increasing T_Flim_. The variation of void density with deposition conditions were also reported elsewhere [31].

**Fig. 6:**
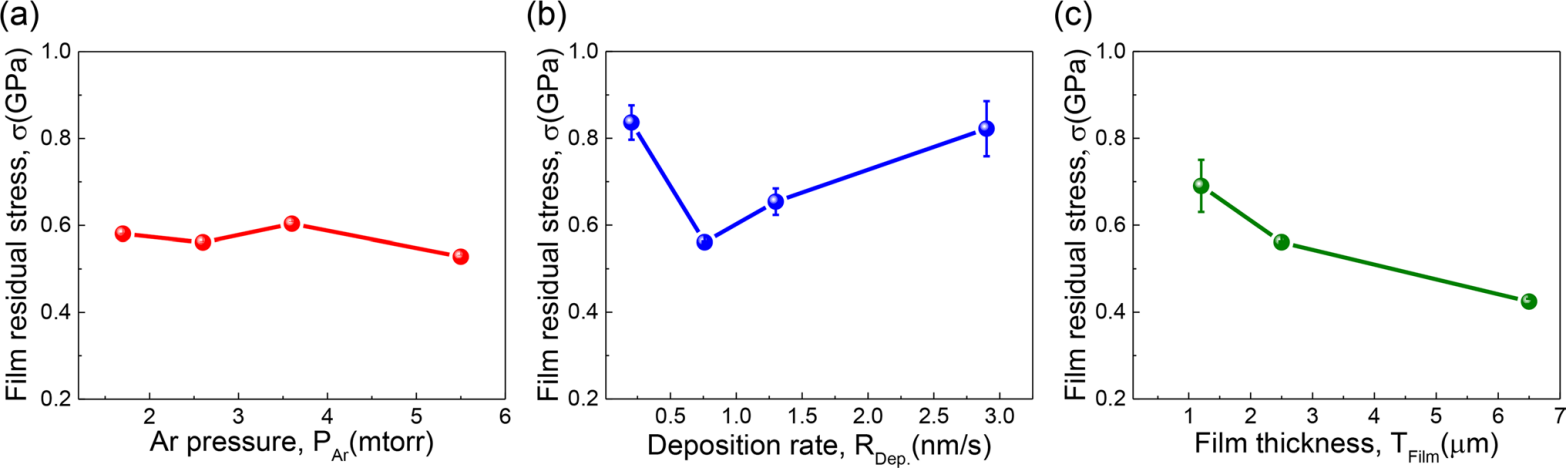
Variation of film residual stress *σ* with increasing **a** Ar pressure P_Ar_, **b** deposition rate R_Dep._, and **c** film thickness T_Flim_

The evolutions of film residual stress *σ* are plotted in Fig. 6. All films have tensile stresses, ranging from 0.4 to 0.9 GPa. Like microstructure features, the residual stress also exhibits a complex dependence on deposition parameters. Detailed statistics on the evolutions of microstructures and residual stresses are summarized in Table 1.

### Type 2: poly-crystalline Cu deposited on Si (111) substrate without seed layer

**Fig. 7:**
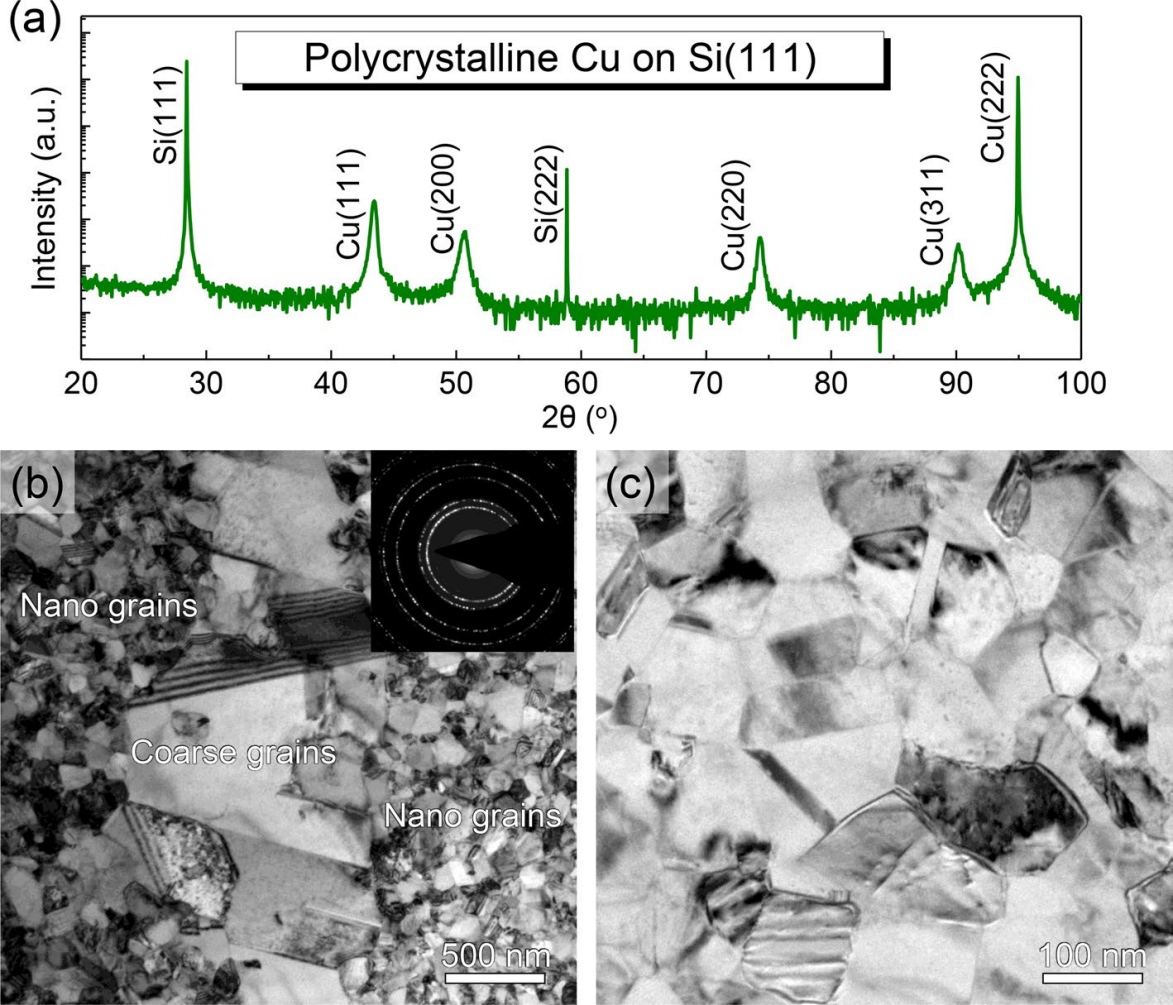
The void-free polycrystalline Cu film directly deposited on Si (111) substrate. **a** 2θ XRD pattern showing diffraction peaks of Cu and Si. **b** The plan-view TEM micrograph of polycrystalline Cu with a bimodal grain size distribution. The inset SAD ring pattern in (**c**) suggests the formation of polycrystalline grains. **c** An enlarged view from the region with small nanograins

The texture and microstructure of sputtered Cu film can be substantially modified by changing the orientation of single crystal Si substrate. Figure 7 illustrates a polycrystalline Cu deposited on HF-etched Si (111). The 2*θ*-scan profile in Fig. 7a shows the existence of several peaks arising from a couple of crystallographic planes of Cu, including (100), (111), (110), and (113). This XRD pattern indicates the formation of polycrystalline grains in as-deposited film. The plan-view TEM micrograph and the inset ring-like SAD pattern presented in Fig. 7b also confirm the formation of polycrystalline Cu. The as-deposited Cu shows a bimodal grain size distribution, with large grain size on the order of 1 μm and small grains less than 100 nm. The enlarged TEM image in Fig. 7c reveals the nanograins are roughly equiaxed without nanovoids at grain boundaries. Additionally, the polycrystalline Cu contains a much lower density of twin boundaries in contrast to the Type 1 NV–NT Cu films deposited on Si (110) (see Sect. 3.1).

### Type 3: nanovoid–nanotwinned Cu (111) deposited on Si (111) with an Ag seed layer

**Fig. 8:**
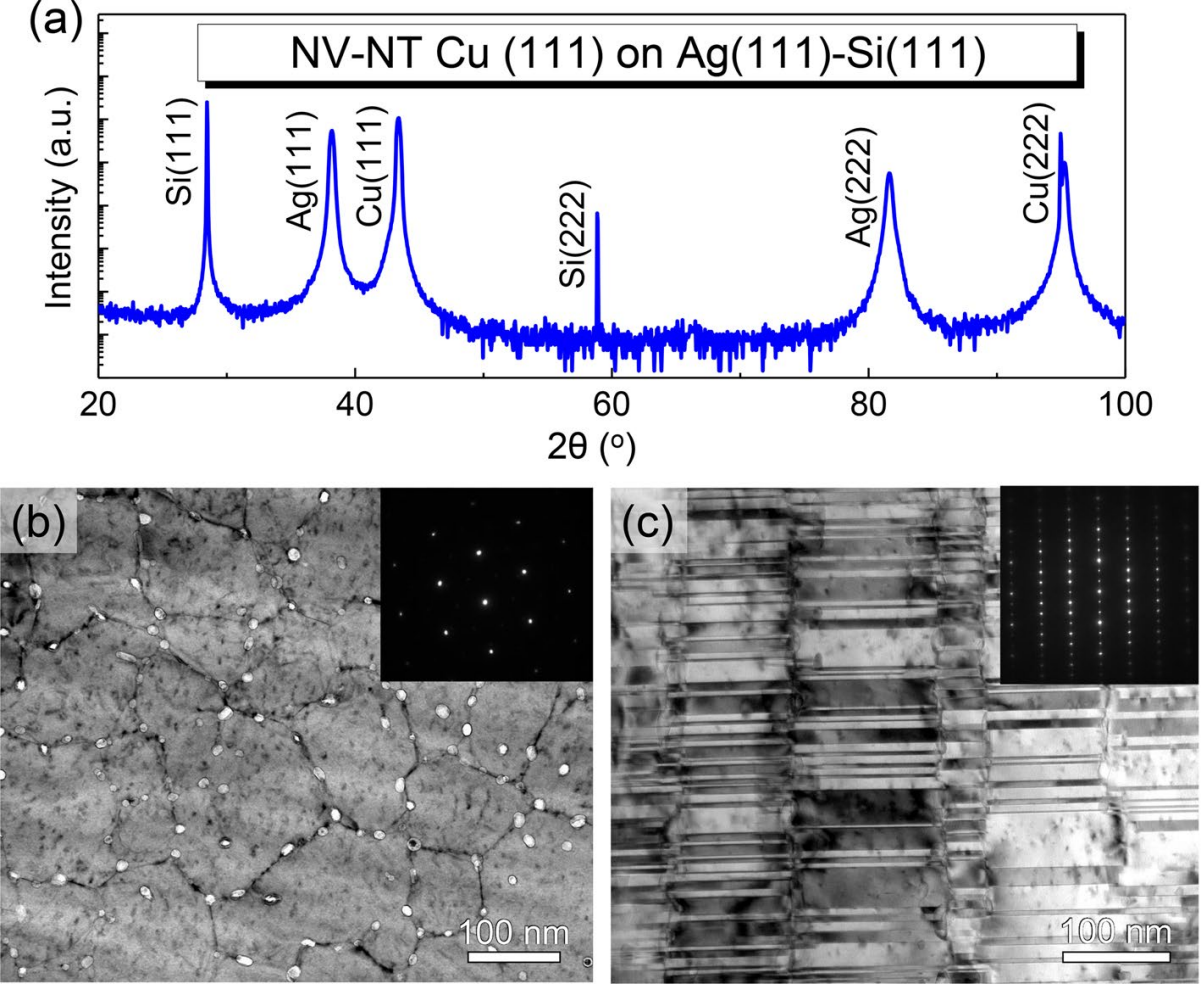
The NV–NT Cu (111) film deposited on Si (111) substrate with an Ag seed layer (~ 200 nm thick). **a** 2theta-scan XRD spectra with (111)/(222) peaks of Cu, Ag, and Si. **b** Plan-view TEM micrograph showing the nanovoids at domain boundaries. **c** Cross-section TEM micrograph showing the nanotwins inside columnar domains

The texture and microstructure of NV–NT Cu film can be regained on Si (111) substrate by adding a seed layer. For instance, Fig. 8 demonstrates an epitaxial Cu (111) deposited on Si (111) with an Ag seed layer (~ 200 nm thick). The XRD 2*θ* scan in Fig. 8a only shows the strong peaks of {111} planes, suggesting the epitaxial growth of Cu (111) on Ag/Si (111). The plan-view TEM micrograph in Fig. 8b shows the formation of nanovoids at domain boundaries, and the inset SAD pattern confirms the growth of epitaxial Cu (111) film. The cross-section TEM micrograph, together with the inset SAD pattern, in Fig. 8c reveals the formation of high-density growth twins in as-deposited NV–NT Cu (111) film.

## Discussion

The microstructural evolution during film deposition has been extensively studied, and the influence of deposition variables (e.g., deposition rate, Ar pressure) can be found in a number of reviews [3, 32–34]. It is generally recognized that a wide range of textures and microstructures can be developed in Cu films depending on preparation methods and conditions [35, 36]. In the current case of sputter-deposited NV–NT Cu (111), however, we found that varying deposition conditions alone can only modify the size, density, and distribution of microstructural features to some extent (see Sect. 3.1). The formation of nanovoids seems to be more strongly dependent on film texture (see Sects. 3.2 and 3.3). For this reason, in the following paragraphs, we will focus our attention on the influence of growth twins on the void formation in epitaxial Cu (111) film.

**Fig. 9:**
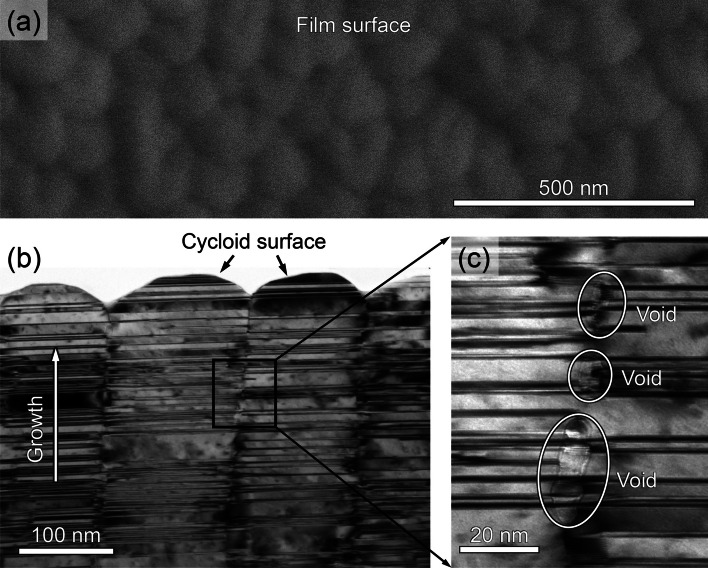
Topography of film growth front of NV–NT Cu (111). The film is labeled as ‘b2_1.3 nm/s’ in Table 1. **a** SEM micrograph of the film surface. **b** Cross-section TEM micrograph of the film growth front with a cycloid surface. **c** Enlarged view of the boxed area in (**b**) demonstrating voids separately aligned along a domain boundary

**Fig. 10:**
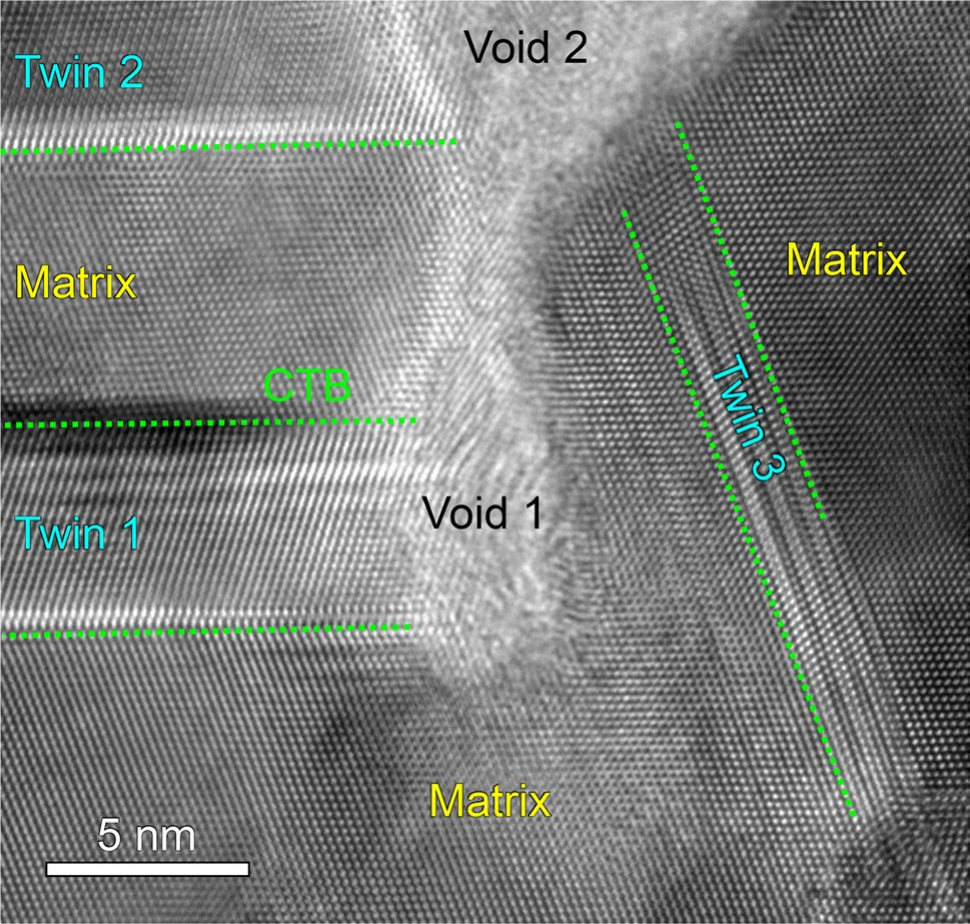
High-resolution TEM micrograph of boundary voids in NV–NT Cu (111)

First, considering that the evolution of film microstructure is intimately related to the film growth process, we carefully inspected the growth front of NV–NT Cu film to obtain some hint of growth kinetics. As shown in Fig. 9a, the NV–NT Cu film surface exhibits ‘island’ configurations when observed from the top-down view. In addition, the cross-section (side view) TEM micrograph in Fig. 9b reveals a cycloid surface profile and a high density of the CTBs underneath the surface. The enlarged view in Fig. 9c shows small voids that are vertically aligned at the domain boundary. Such surface characteristics suggest that the boundary voids may form as columnar domains come into contact with each other. To reveal where the voids are nucleated along the columnar boundary, we further performed high-resolution TEM analysis. As shown in Fig. 10, there are three twins (Twins 1–3) separated by CTBs in the matrix. Also, there are two boundary voids, Voids 1 and 2 formed at the ends of Twin 1 and Twin 2, respectively. Between the voids, however, the matrix is almost joined with little spacing. It seems that the voids are likely to nucleate at the regions where the twin and matrix impinge. Based on these observations, we finally explain the growth of epitaxial NV–NT Cu (111) within the framework of island coalescence model [37]. According to this model, the film grows by the nucleation and coalescence of discrete islands when they come to impinge on each other [38, 39]. Also, this model predicts a large elastic strain in the film, which is in qualitative agreement with our experimental results in Fig. 6.

**Fig. 11:**
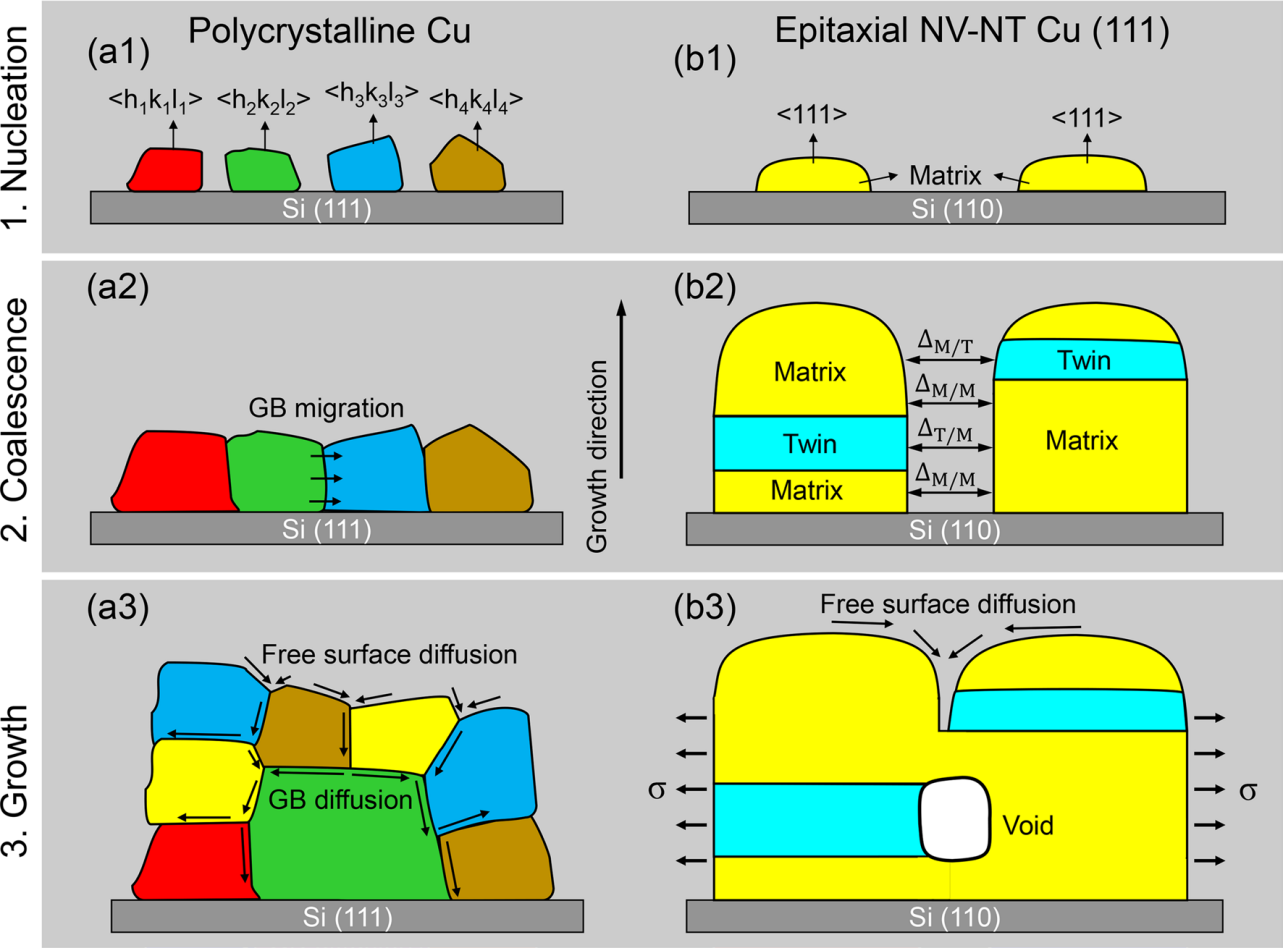
Two-dimensional schematic representation of crystallite nucleation, coalescence, and growth in sputtered Cu films. **a1**–**a3** Formation of void-free polycrystalline Cu film on Si (111) substrate due to GB diffusion and migration. **b1**–**b3** Void formation mechanism through crystallite coalescence in epitaxial NT Cu (111) film on Si (110) substrate

Following the island model, we can attribute the void formation to the increased energy barrier against coalescence when growth twins are present inside islands. The underlying mechanisms are illustrated schematically in Fig. 11. Here, we are first concerned with the microstructure of a polycrystalline Cu and attempt to understand why it is void-free. As shown in Fig. 11a1, when Cu is deposited onto Si (111) substrate, crystallites with random orientations *h*_*i*_*k*_*i*_*l*_*i*_ (*I* = 1, 2, 3, 4…) are initially nucleated. During deposition, these individual crystallites grow continuously until they coalescence into a continuous polycrystalline film, as schematically shown in Fig. 11a2. At this point the residual tensile stress might be very high, and some voids might also be formed due to the shadowing effects [40]. At the later stages of film growth, however, the stress can be relaxed, and the voids can be removed by incorporating additional adatoms into grain boundaries through fast diffusions along surface—grain boundary network, as shown in Fig. 11a3. Meanwhile, grain boundary migration and secondary grain growth could be caused by local substrate heating. This process results in a bimodal grain size distribution, as observed in Fig. 7 or reported elsewhere [35, 41].

In comparison, when Cu is deposited onto Si (110), or onto Si (111) with an Ag seed layer, the individual crystallites are all preferentially <111>-orientated, as shown in Fig. 11b1. The orientation relationship could be determined by the geometrical lattice match rule [42]. As {111} is the twin plane for an FCC metal, and the growth twins are favored to nucleate on this plane for Cu due to its low stacking fault energy (45 mJ/m^2^) [43, 44]. The crystallites, therefore, are composed of fine growth twins, as shown in Fig. 11b2. Although these twins and their matrix are oriented the same along the film growth direction, they have a large rotation angle (60°) in the film plane (twin plane). Hence, as the NT crystallites snap together on side walls, incoherent twin boundaries (ITBs) are expected to form at the intersections where twins meet the matrix, while at other locations where matrix faces matrix (or twin faces twin), no grain boundaries would form. The maximum gap size between adjoining crystallites can be estimated based on a simple energy criterion that the reduction of surface energy balances the increase of boundary energy and elastic energy [37]. For the twin-matrix segments, the maximum gap size $\Delta_{T - M}$ is described as [39]

2 $$\Delta_{T - M} = \left[ {2D\left( {2\gamma_{sv} - \gamma_{ITB} } \right)\frac{1 - \upsilon }{E}} \right]^{1/2}$$

where *D* is the crystallite (domain) size, $E$ is the Young’s modulus, $\upsilon$ is the Poisson’s ratio, $\gamma_{sv}$ is the surface energy, and $\gamma_{ITB}$ is the incoherent twin boundary energy. For the matrix–matrix (or twin–twin) segments, the maximum gap size $\Delta_{M - M}$ (or $\Delta_{T - T}$) is in the form of

3 $$\Delta_{M - M} = \left[ {4D\gamma_{sv} \frac{1 - \upsilon }{E}} \right]^{1/2}$$

Combining Eqs. (2) and (3), we can conclude that $\Delta_{M - M} > \Delta_{T - M}$, indicating that the joining between matrix and matrix (or twin and twin) is energetically favorable over the joining between twin and matrix. As a result, a void tends to nucleate at the twin–matrix intersection region when the regions below and above are ready to join (snap) together, as illustrated in Fig. 11b3. Upon nucleation, the void will be buried beneath the surface and is cut off from the surface diffusion. However, whether the buried void can remain intact thereafter also depends on the bulk diffusion. Note that, unlike the polycrystalline Cu that is composed of regular grain boundaries, the NT Cu (111) is dominated by CTBs, the special low-energy and high-coherent boundaries along which the diffusion is limited. Therefore, the buried void is able to survive the film growth, insomuch as the net diffusion into it is restricted.

The foregoing mechanism of void formation suggests that the NT Cu (111) film residual stress cannot be relaxed timely by incorporating additional adatoms. This is consistent with our experimental measurements. As shown in Fig. 6, all the sputtered NV–NT Cu have a large tensile stress ranging from 0.4 to 0.9 GPa. It has been pointed out that tensile stress might promote void nucleation and growth [45]. Consequently, more nanovoids are expected to form along domain boundaries provided that twins are present in the growing domains, as confirmed by our high-resolution TEM micrograph in Fig. 10. This mechanism also suggests that the void size and twin thickness should be comparable, as the formation of nanotwins precedes the nucleation of nanovoids. Indeed, Figs. 5a–c demonstrate that the twin size and void size are similar in most of the epitaxial NT Cu films regardless of the deposition conditions. The systematic studies presented here thus provide a practical method to manufacture NV–NT Cu. The discovery reported in epitaxial Cu may be applicable to other epitaxial NT metals.

## Conclusion

Polycrystalline and epitaxial Cu thin films were synthesized by direct current magnetron sputtering deposition technique. The texture and microstructure of as-deposited films can be tailored by varying deposition conditions, changing orientation of Si substrate, or adding an Ag seed layer. The polycrystalline Cu film exhibits a typical bimodal distribution of grains with almost no nanovoids. In comparison, for the epitaxial Cu (111) films grown on Si (110) or on Si (111) with an Ag seed layer, their microstructures are characterized by high-density nanotwins and nanovoids. The nucleation of nanotwins inside columnar domains can be attributed to the low stacking fault energy of Cu, while the nucleation of nanovoids at domain boundaries is caused by the high energy barrier at the twin-matrix intersections. The formation of these nanovoids in the epitaxial Cu films can be ascribed to the cutoff of surface diffusion and the restriction of bulk diffusion. Consequently, the nanovoid formation mechanism in NT Cu can be rationalized based on the proposed island coalescence model. This study suggests that texture and twin boundaries can play an important role in tailoring the formation of nanovoids in NT metals.

## Data Availability

Data in the current study are available from the corresponding author, Cuncai Fan, upon reasonable request.
